# Supplementary material for: Patterns of Intron Gain and Loss in Fungi
Source: PLoS Biol. 2004 Nov 30;2(12):e422. doi: 10.1371/journal.pbio.0020422 (PMC532390; doi:10.1371/journal.pbio.0020422)
Supplement: Table S1 — Also available at http://genes.mit.edu/NielsenEtAl/. (4.3 MB ZIP). [file pbio.0020422.st001.zip › NielsenEtAl/html/235.html]

AN2939.1.NCU03217.1.MG03684.1.FG09158.1


```
 CLUSTAL W (1.82) Multiple Sequence Alignments - Introns Inserted


Sequence 1: NCU03217.1	548 aa
Sequence 2: MG03684.1	601 aa
Sequence 3: FG09158.1	550 aa
Sequence 4: AN2939.1	543 aa
Alignment Length: 616 aa
Number Identitical Residues: 248 aa
Alignment Score (without introns) 11758


MG03684.1 	MPN1LLISRDLGAELNPPLSNDDAISNDSADLPSHHFGICLTSEGHPSSVMSASASVARR
NCU03217.1	---~----------------------------------------------MSASSTVARR
FG09158.1 	---~----------------------------------------------MSASSIVARR
AN2939.1  	---~----------------------------------------------MSVSMRSARL
          	                                                  **.*   ** 

MG03684.1 	AVVMNPLVGNG1RA~LPRSVLRASQAHLGRH--SRNLALQNPAIALLIPVRSLSTD-THT
NCU03217.1	ALVVNPFLGAG1VA~LPRQGLLAASTLLARN--SRHIMLNNQTSALVFPVRPITTSGTST
FG09158.1 	AIARNPFMGNA1RA~LSRG---VTPAVLGRHV-SRHAQIP--AISLLIP-RSMSTD--HH
AN2939.1  	VRSNPVLRPNS~IA1RQRYAGAFGAAATGLRFNSRNFPSQVTALAILVPKRGYATE-QST
          	.     :   .  *   *       :  . . .**:      : :::.* *  :*.    

MG03684.1 	SGRGVPSVPPPGFNAEKAKNPLPKEEKPNDNAG--KAPVPADSAAVKPTAVAEASENSAS
NCU03217.1	HGSGPPAGPPPGFNAEEAKKPLPREAATLPVKS--KTIAGEVQEVTKKTTEALAG-TEAS
FG09158.1 	RPDPSSSGPPPGFNAEQAKKPLPKDSTIVAKKEDKKLDKTVNGSAILKPADAAASPNAIN
AN2939.1  	NTSGPSNLPPPGFNAEQAKKPISVDQAQPASAKANQDTIPKGEVSVQSQNAQSTSKESGL
          	     .  ********:**:*:. :         .:                 :.     

MG03684.1 	LTELAADK-AAEKKEEPK--KEEKKLTLGQKIKKEAQHYWDGTKLLAAEVKISSRLALKM
NCU03217.1	QSQLAEQKQAGAEKSEGK--KEEPKLTLKQKIKKEAQHYWDGTKLLAAEVRISSRLAIKM
FG09158.1 	QEVVATNEGVMEKADAAQKAKEEKKLTLWEKVKKEAHHYWDGSKLLVAEVKISWRLALKM
AN2939.1  	ASKSVAEDKDKKAVEEPK--KESKKLTIGQKIKKEIQHYWDGTKLLATEVRISSRLALKM
          	    . :.      .  :  **. ***: :*:*** :*****:***.:**:** ***:**

MG03684.1 	AAGYELTRRESRQ~LQRTVQDLGRLVPFSVFIIVPFAELLLPVALKLFPNLLPSTYEGQK
NCU03217.1	AAGYELTRRENRQ~LRRTVQDLGRLVPFSMFVIVPFAELLLPVALKLFPNMLPSTYEEQK
FG09158.1 	AAGYELTRRENKQ~LQRTVQDLGRLVPFSVFIIVPLGEALLPLALKLFPNMLPSTFEGQK
AN2939.1  	AGGYELSRREHRQ0LKRTVTDLGRLIPFSMFVIIPFAELLLPVALKLFPNLLPSTYEGKS
          	*.****:*** :* *:*** *****:***:*:*:*:.* ***:*******:****:* :.

MG03684.1 	SRDKKTNALRATRKEVSGFLRQTLKETGLPVTAATTQRDEFTNFFRKLRSTGEKPTAEDV
NCU03217.1	SKDKKASTLRATRKEVSDFLRKTMKETGLPLTQVTAQKEEFSNFFRKVRSTGEKPTAQDV
FG09158.1 	SKEAKATILRSTRKEVSEFLRQTLGEG-LPLSQATTQKEEFSNFFRKVRATGETPTAQDV
AN2939.1  	AREKKALSLSSTRKEVSTFLKNTLKESGLPVTAASVKNDEFAEFFKKIRSTGETPSAEDV
          	::: *:  * :****** **::*: * .**:: .:.:.:**::**:*:*:***.*:*:**

MG03684.1 	IRVCKIFRDDLTLDNLSRPQLVSMCRYLNLNTFGTDMMLRYQIRHRMRQIKRDDKAISYE
NCU03217.1	IKVCKIFKDDLTLDNLSRPQLVSMCRYLNLNTFGTDMMLRYQLRHRMRQIKRDDRAISYE
FG09158.1 	IKICKAFRDDLTLDNLSRPQLVSMCKYMNLSTFGTDMMLRYQIRHRMRQIKRDDKAISYE
AN2939.1  	IKVCKIFKDDLTLDNLSRPQLVGICKYMNLNTFGTDAMLRYNIRHRMRQIKRDDRAIFYE
          	*::** *:**************.:*:*:**.***** ****::***********:** **

MG03684.1 	GVDSLTVAELQTACQSRGIRTHGISPARLREDLQTWLDLRLKDGVPSTLLVLSSAYMYSQ
NCU03217.1	GIETLSVAELQVACASRGIKSYGVSPARLREDLQTWLDLRLREGVPSTLLVLSNAYMYGQ
FG09158.1 	GVDSLTVSELQAACAARGIRTHSVSPARMRNDLQTWLDLRLKEGVPSTLLVLSNAYMYGQ
AN2939.1  	GIDSLSVPELQMACASRGIRTHGVSPARLRDDLSQWLDLRLKQGVPSTLLVLSNAYVYAQ
          	*:::*:*.*** ** :***:::.:****:*:**. ******::**********.**:*.*

MG03684.1 	ASSSAALEDGEVSGQIEALTGVLSSIPEELFHEIELEVHNAEGAATNKQRLEVLKEQQEL
NCU03217.1	T-------DGEVSSQIEALTGVLSSIPEELFHEIELEVHNAEGAATNKQRLEVLKEQQEL
FG09158.1 	G-------SGEGSGQVEALIGVMSAIPEELYHEIELEVHSAEGAATNKQRLEVIREQQEL
AN2939.1  	GG-----KEAEMSSQIESLQAVLSSIPEELFHEIELEVHNAEGAATNKQRLEVIKEQQEL
          	 .     ...* *.*:*:* .*:*:*****:********.*************::*****

MG03684.1 	IEEENEQNQGNEQTGLATPRDVDDIDEKDEREMQAAKQAATEELGGVEKQQVGDMVEAEQ
NCU03217.1	IDEELQQDQENQKTGFATPRDTEDIDEDHERHIQAKADGIEKAQVSEAVDAEQEALTAAR
FG09158.1 	IEDEAEQDQASQSSGFATPRDTDDIDEKEERLAQAQAEGLGRKQVSEMVEAETELAKAAE
AN2939.1  	IEEENQQNSENEEKGVAAPKDTENIDEDHKYETTQSGEASEAMQEGEKAEKDAEPAVQEK
          	*::* :*:. .:..*.*:*:*.::***..:       :.      .   :   :     .

MG03684.1 	----ITLA--EKTAKKSEK-
NCU03217.1	----VEQAGEQKSEKTTEK-
FG09158.1 	SARLLEREIQASSGSSKEQK
AN2939.1  	--------------KDTK--
          	              . .:
```
